# *Pelargonium sidoides* Root Extract: Simultaneous HPLC Separation, Determination, and Validation of Selected Biomolecules and Evaluation of SARS-CoV-2 Inhibitory Activity

**DOI:** 10.3390/ph15101184

**Published:** 2022-09-23

**Authors:** Manal A. Alossaimi, May A. Alzeer, Fatma M. Abdel Bar, Mai H. ElNaggar

**Affiliations:** 1 Department of Pharmaceutical Chemistry, College of Pharmacy, Prince Sattam Bin Abdulaziz University, Al-Kharj 11942, Saudi Arabia; 2 Department of Pharmacognosy, College of Pharmacy, Prince Sattam Bin Abdulaziz University, Al-Kharj 11942, Saudi Arabia; 3 Department of Pharmacognosy, Faculty of Pharmacy, Mansoura University, Mansoura 35516, Egypt; 4 Department of Pharmacognosy, Faculty of Pharmacy, Kafrelsheikh University, Kafrelsheikh 33516, Egypt

**Keywords:** COVID-19, HPLC-UV, quality control of Umckaloabo, *Pelargonium sidoides* root extract, umckalin

## Abstract

This study aimed to establish a validated HPLC-UV analytical method for the determination of gallic acid, catechin, scopoletin, and umckalin in phytoformulations containing *P. sidoides*. Also, to assess the anti-SARS-CoV-2 effect of *P. sidoides* and these biomolecules in vitro. An HPLC-UV method was developed and verified by testing the commercial forms, Kalobin^®^ and Umca^®^. It revealed low detectable scopoletin and high umckalin levels. *Pelargonium sidoides* exhibited a significant reduction of SARS-CoV-2-induced cytopathic effect in Vero E6 cells (IC_50_ 13.79 μg/mL and selectivity index, SI 6.3), whereas scopoletin showed a remarkable anti-SARS-CoV-2 activity with better selectivity (IC_50_ 17.79 μg/mL and SI 14.22). An in-silico prediction of the drugability indicated that the studied biomolecules are under the acceptable norms of Lipinski’s rule, water-soluble, and showed high GIT absorption and bioavailability. Docking study towards the essential molecular targets for viral replication and entry of SARS-CoV-2 indicated good binding affinity of scopoletin (−6.4 Kcal/mol) towards the interface region between the SARS-CoV-2 spike protein RBD and the ACE2 surface receptor indicating the probability of interference with the viral entry to the human cells and showed H-bonding with His-41 in the active site of the main protease which may explain its high antiviral activity.

## 1. Introduction

Medicinal plants have been used by humans for thousands of years and they are the basis for today’s advanced medications. One of the commonly used medicinal plants indigenous to South Africa is *Pelargonium sidoides* DC. (Geraniaceae), also known as Umckaloabo. The root extract of the perennial flowering plant (EP_S_ 7630) has been used by the local population for a very long time to treat a variety of symptoms, such as dysentery, diarrhea, hepatic complaints, wounds, cold, and various infections of the respiratory tract, including tuberculosis [1]. The major constituents and/or extracts of *P. sidoides* (Umckaloabo) were reported to exhibit antibacterial and antiviral activities against several bacteria and respiratory viruses [2,3]. Umckaloabo was recently suggested as a promising adjuvant treatment for the pandemic disease, COVID-19 [4]. However, more research is required to fully investigate the efficacy of this plant against SARS-CoV-2 and the compound (s) that could be responsible for this activity.

Several studies have examined the composition of the plant and found a plethora of coumarins and phenolic metabolites [1]. As reported before, some highly oxygenated coumarins are among the factors governing the pharmacological efficacy of the *P. sidoides* plant [5]. These include 7-hydroxy-5,6-di-methoxycoumarin (umckalin), 6-hydroxy-7-methoxycoumarin (scopoletin), and 6,8-dihydroxy-5,7-dimethoxycoumarin, in addition to gallic acid, and flavonoids (such as catechin) [5,6]. Recently, the chemical profile of *P. sidoides* root was characterized by high-resolution MS with the identification of 33 compounds, including phenolic acids, coumarins, flavonoids, polyphenols, vitamins, and nucleotides [7].

The pharmacological activities of herbal medications cannot be attributed to a single compound within the medication but rather to a multitude of compounds [8]. Hence, the development of systematic quality control systems and validation methods is essential to ensure that the produced herbal medicines are both safe and efficient in achieving the targeted action [9]. The use of the *Pelargonium* spp., including *P. sidoides* DC. and/or *P. reniforme* Curt. is accepted by the European Pharmacopoeia without defining specific differentiation parameters [10]. It is worth noting that umckalin was only reported in *P. sidoides* rather than *P. reniforme* [11,12]. The control of the *P. sidoides* root and its pharmaceutical preparations is usually performed through a pharmacopeial method based on the determination of tannins [10,13], which is not satisfactory for the quality control of the herbal medications and the corresponding formulations.

**Figure 1:**
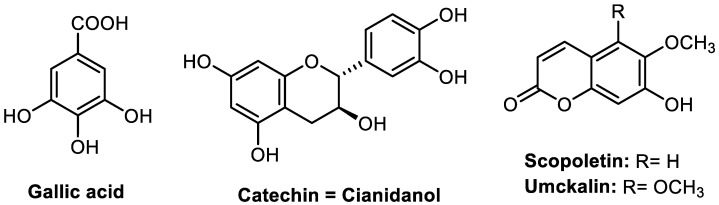
Chemical structures of the four studied biomolecules of *Pelargonium sidoides* in phytopreparations.

In this study, four main representative biomolecules, including one phenolic acid (i.e., gallic acid), one flavan derivative (i.e., catechin), and two coumarins (i.e., scopoletin, and umckalin) have been selected for the HPLC analysis and biological evaluation of this plant’s phytopreparation. The chemical structures of these biomolecules represent the main phytochemical classes of *P. sidoides* which are coumarins and phenolic compounds as depicted in Figure 1. The separation and determination of these biomolecules from other plants have been extensively studied in the literature. Numerous analytical methods have been used for gallic acid [14,15], catechin [16,17], and scopoletin [18,19]. However, to the best of the authors’ knowledge, only one analytical method has been reported for the determination of umckalin, through an HPLC-RP method using a C18 column, an isocratic solvent system of acetonitrile-water (45:55, *v*/*v*) flowing at a rate of 0.75 mL/min, and by UV detection at 330 nm [20]. Other analytical procedures suitable for the quantitative and qualitative determination of umckalin can be found in [7,21]. To date, no analytical method has been proposed to simultaneously separate or detect these four important biomolecules by HPLC. In addition, neither validation nor optimization has been described for the analytical methods reported in the literature.

Hence, the objective of this work is to develop a novel validated HPLC method for the determination of the four suggested biomolecules (*viz*., gallic acid, catechin, scopoletin, and umckalin) in commercial solutions and in tablet formulations containing *P. sidoides* (Umckaloabo) extracts. Also, to evaluate and compare the efficiency of different sample preparation procedures in extracting the studied biomolecules. As well as to investigate the antiviral activity of *P. sidoides* root extract and the investigated biomolecules against SARS-CoV-2. The physicochemical and pharmacokinetic properties of the studied biomolecules were investigated in silico to predict their potential drugability and effectiveness. Moreover, to get insight into the binding interactions of these biomolecules against the essential molecular targets for viral replication and entry of SARS-CoV-2, a computational docking study was performed. The docking study involved several targets, including the papain-like protease (PLpro), the main protease (Mpro), RNA helicase (nsp13), and RNA-dependent RNA polymerase (RdRp, nsp12) that are essential for viral replication [22,23,24,25,26,27]. Also, the ability of these compounds to inhibit viral entry to the human cells via interfering with the interaction between the SARS-CoV-2 spike protein and the human angiotensin-converting enzyme 2 (ACE2) receptors was virtually examined using the docking study [28,29].

## 2. Results and Discussion

The quality assurance of phytomedications starts from the correct and reliable identification of the raw materials. Suitable identification and detection procedures must be able to distinguish between multiple active compounds and related species. To complicate things further, a major challenge when dealing with this type of medication is the noticeable variation in the concentrations of the active compounds between different instances of the same species of plants. This variation is attributed to several important factors, including the soil type, geographical location, climate, age, time of harvesting, storage condition… etc. Analytical methods, such as chromatography and spectroscopy can be used to achieve proper quality control (QC) in herbal medications. It is generally advisable to use combinations of chromatographic and spectroscopic methods to overcome their limitations [11].

### 2.1. HPLC Method Development and Optimization

**Figure 2:**
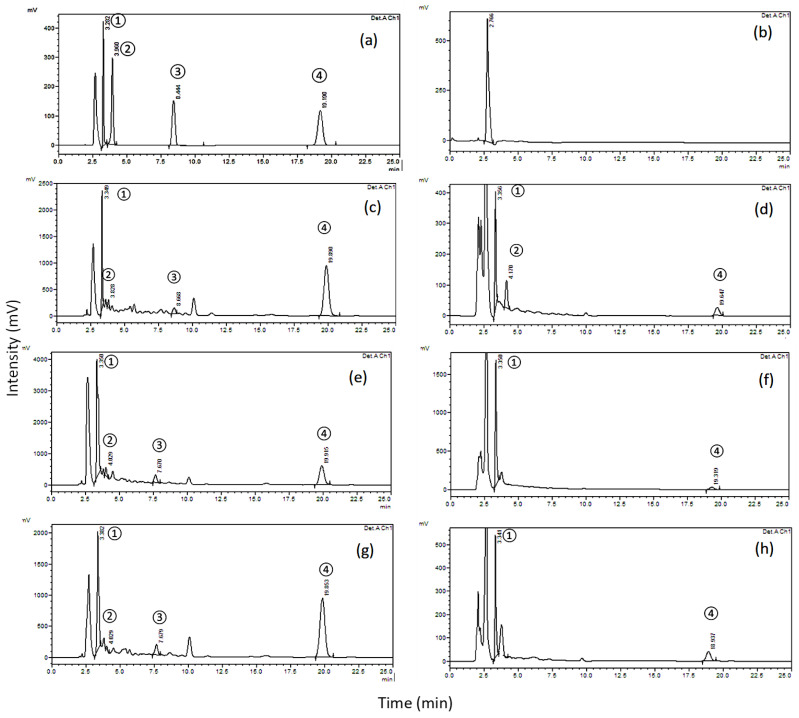
HPLC-RP chromatograms (at 210 nm) of: (**a**) 0.16 µg/mL standards mixture of (1) gallic acid, (2) catechin, (3) scopoletin, and (4) umckalin obtained at retention times of 3.3, 3.96, 8.4 and 19.2 min, respectively; (**b**) Blank (methanol); (**c**) Kalobin^®^ solution extracted with ethyl acetate; (**d**) Kalobin^®^ solution extracted with methanol; (**e**) Umca^®^ solutions extracted with ethyl acetate; (**f**) Umca^®^ solutions extracted with methanol; (**g**) Umca^®^ tablets extracted with ethyl acetate; and (**h**) Umca^®^ tablets extracted with methanol.

An extensive survey of the literature related to our studied four biomolecules, gallic acid, catechin, scopoletin, and umckalin, was carried out to identify the known physical and chemical properties of these compounds [30,31,32,33]. By varying one parameter at a time while keeping the remaining parameters constant, an optimization of simultaneous chromatographic separation conditions proceeded, including the detection wavelength, mobile phase, stationary phase, and sample preparation procedure. A series of trials were carried out with discrete values of the acetonitrile to water ratio and with pH values of 3, 8, and 9 and two brands of columns (Hypersil BDS column (250 × 4.6 mm, 5 µm particle size) (Thermo Scientific Inc., Bremen, Germany) and Waters XBridge^®^ C18 column (200 mm × 4.6 mm, 5 µm particle size, Milford, MA, USA). The most suitable chromatographic conditions for this study were found to be acetonitrile to water ratio of 20: 80 *v*/*v* (pH 3), 1 mL/min flow rate, 20 µL injection volume, 25 min run time, using the Waters XBridge^®^ C18 column (200 mm × 4.6 mm, 5 µm particle size, Milford, MA, USA) occupied at 30 °C, and a detection wavelength of 210 nm. When the four compounds under the study were eluted simultaneously, the chromatogram showed symmetrical peaks within a minimal analysis time. The retention times of gallic acid, catechin, scopoletin, and umckalin were found to be 3.3, 4.0, 8.4, and 19.2 min, respectively. The results are depicted in Figure 2a. An additional peak appears at 2.5 min. This peak is the solvent front which was coming from the solvent that was used to dissolve the reference standards (methanol) as shown in the blank run in Figure 2b.

### 2.2. Method Validation

The proposed separation and detection method was validated by using the validation parameters, including specificity, linearity, detection and quantitation limits, precision, and robustness as mentioned in the material and methods section according to the guidelines of the International Council for Harmonisation (ICH) [34].

#### 2.2.1. Specificity

The tincture samples were exposed to acid or alkali hydrolysis to produce potential degradation products. The spectral purity of the biomolecules was then compared and used to evaluate the specificity and selectivity of the method [35]. It was found that the method was specific and selective.

#### 2.2.2. Linearity

The obtained calibration curves (Figure S1) were interpolated by means of the least-squares linear regression (LSLR) method [36]. To test the linearity of the proposed method, five or six distinct concentrations were considered for each standard as detailed in Table 1. The concentrations of gallic acid and catechin were taken in the range of 0.2–1 µg/mL, while the scopoletin and umckalin concentrations were set within the range of 0.1–1 µg/mL. For each concentration, three different samples were injected. The algebraic mean of the resulting peak areas was calculated and recorded. The accuracy of the regression process was identified by means of the coefficient of determination ($R^{2}$), which had values of 0.9995, 0.9999, 0.9998, and 0.9995 for gallic acid, catechin, scopoletin, and umckalin, respectively. Since all values were extremely close to 1, the proposed method is considered to have good linearity. The obtained regression line equations for the gallic acid, catechin, scopoletin, and umckalin biomolecules were y=129747x+1655.6, y=164167x+2039.8, y=162562x+2223.7, and y=188550x+3047, respectively.

#### 2.2.3. Detection and Quantitation Limits

When assessing the sensitivity of any method, it is important to quantify its limit of detection (LOD) and limit of quantitation (LOQ). The LOD refers to the smallest concentration of the measured compound that can be detected by the studied method to a certain high confidence. The LOQ is the smallest concentration that can be determined with a reasonable level of precision and accuracy [36]. Both limits were calculated from the regression equations shown in Table 1 and the resulting LOD and LOQ were: 0.024 and 0.073 µg/mL for gallic acid; 0.012 and 0.036 µg/mL for catechin; 0.012 and 0.036 µg/mL for scopoletin; and 0.018 and 0.055 µg/mL for umckalin, respectively. The equation used to calculate these values is illustrated below:

LOD = 3.3 S/b

LOQ = 10 S/b

S = Standard deviation of the response
b = Slope of the calibration curve

#### 2.2.4. Precision and Accuracy

To identify the accuracy and precision of the proposed analytical procedure, three replicates of each QC sample concentration were considered along with the five calibrators. Recall that the concentrations of the QC samples were chosen as 0.2, 0.3, and 0.5 µg/mL (low, medium, and high). As shown in Table 2, the accuracies of the proposed method concerning gallic acid, catechin, scopoletin, and umckalin were found to be 100.89%, 99.57%, 100.03%, and 100.51%, respectively. Table 2 shows the precision of the method for all four biomolecules. The relative standard deviation for all samples was below 1%.

Our method showed lower LOD and LOQ for gallic acid [14,15], catechin [16], scopoletin [7,18] compared to other detection methods. However, better sensitivities were reported for catechin [17] and scopoletin [19] due to the highly sensitive detectors (i.e., UHPLC–MS/MS and fluorescence detectors, respectively). Although Panara, et al. calculated the LOD of gallic, catechin, and scopoletin, no values were calculated for umckalin [7]. In our study, umckalin showed almost comparable LOD to the previously reported method using a DAD detector [20].

#### 2.2.5. Robustness

The robustness of the proposed method was investigated. It was found that temperature variations did not have an impact on the analytical results. However, the rest of the conditions, including the brand of the column, the flow rate of the mobile phase, and its composition all had a significant influence on the quantifications. This resulted in some small changes in the retention times of the detected peaks, and consequently, the integration was impacted. In addition, when the prepared samples were kept at a temperature of 4 °C, they remained stable for at least 4 days. To further investigate the stability of the standards, calibration solutions were injected into the HPLC system at 30-day intervals after being stored at 4 °C. The obtained peaks were almost identical and could be superimposed. This indicates that the calibration solutions were stable.

### 2.3. Determination of the Four Biomolecules in Kalobin^®^, Umca^®^ Solutions and Umca^®^ Tablets

The measured average concentration percentages of the four biomolecules in different dosage forms, namely Kalobin^®^ and Umca^®^ solutions and the Umca^®^ tablets are shown in Table 3. The obtained results were satisfactory, and no interference was observed. Figure 2c–f depicts the chromatograms corresponding to the Kalobin^®^ and Umca^®^ solutions, while Figure 2g,h shows that of the Umca^®^ tablets. In these figures, two extraction solvents are shown: ethyl acetate, Figure 2c,e,g, and methanol, Figure 2d,f,h. It can be observed that ethyl acetate was efficient at extracting all biomolecules, whereas methanol was only suitable for the extraction of gallic acid, catechin, and umckalin and poorly detects scopoletin. Hence, with the appropriate choice of the extraction solvent, all four biomolecules can be efficiently detected in Kalobin^®^, Umca^®^ solutions and Umca^®^ tablets using the proposed analytical method for assay. The proposed HPLC analytical method using ethyl acetate extraction in sample preparation showed high contents of gallic acid and umckalin. However, it revealed lower detectable levels of catechin and scopoletin, with the latter maximum concentration of 27.19 μg/mL in Umca^®^ tablets, Table 3 and Figure 2.

### 2.4. In Vitro Inhibitory Activity against SARS-CoV-2

**Figure 3:**
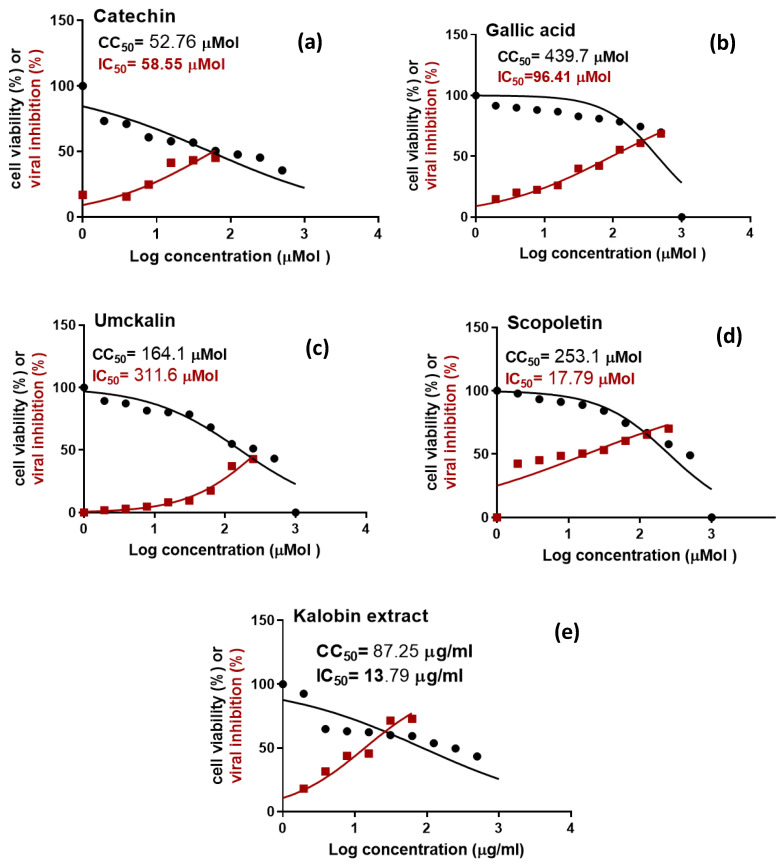
Dose-response curves of the tested compounds: (**a**) Catechin, (**b**) Gallic acid, (**c**) Umckalin, (**d**) Scopoletin, and (**e**) Kalobin extract showing their 50% Cytotoxic concentration on Vero E6 cells (CC_50_) and their 50% Inhibitory concentration (IC_50_) against SARS-CoV-2 on Vero E6 cells.

The in vitro antiviral screening assay indicated that *P. sidoides* root extract (Kalobin) has promising antiviral activity against SARS-CoV-2 (Table 3 and Figure 3) and the concentration required to cause a 50% fall in the viral-induced cytopathic effect (IC_50_) was found to be 13.79 μg/mL. On the other hand, the concentration required to cause a 50% growth inhibition of the normal Vero E6 cells (CC_50_) was found to be 87.25 μg/mL. Thus, the *P. sidoides* root extract showed a selectivity index (SI) for antiviral activity relative to cellular toxicity equals 6.3. The obtained results were in agreement with those published before on rhinovirus 16 (RV16), which showed that *P. sidoides* reduces the infection and improves the survival rate of the host, human bronchial epithelial cells (hBEC) [37]. Scopoletin showed the highest antiviral activity among the tested biomolecules with a lower IC_50_ (17.79 μM) than the standard references chloroquine and hydroxychloroquine (IC_50_ of 22.7 μM and 32.8 μM, respectively) [38]. It also showed a SI of 14.22 which was comparable to that of chloroquine and hydroxychloroquine (SI 16.64 and 10.85, respectively) [38]. Scopoletin was previously suggested as a potential antiviral agent against SARS-CoV-2 based on its in-silico binding interactions with the main viral protease (Mpro) [39]. Although many studies correlated the therapeutic applications of *P. sidoides* root extract to its main biomolecule (umckalin) [40], however, umckalin showed a poor antiviral effect against SARS-CoV-2 with an IC_50_ value of 311.6 μM. These results were in harmony with those published by Trun et al. (2006) against Leishmania-induced cytopathic effect in RAW 264.7 cells, which supports our findings as umckalin appears to be inactive as an antimicrobial drug [41]. It could be concluded that the antiviral activity against SARS-CoV-2 can be attributed to scopoletin. In addition, further pharmacological studies are still required to identify the other phytochemicals responsible for the immunological and antimicrobial activities of the *P. sidoides* plant.

### 2.5. In Silico Investigation of the Physicochemical and Pharmacokinetics Properties Using SwissADME Online Platform

The physicochemical and pharmacokinetic properties affecting absorption, distribution, metabolism, and excretion were computationally evaluated to provide an understanding of the in vivo antiviral activity of the tested compounds. Results (Table S2) articulated that the four studied biomolecules are under the acceptable norms of Lipinski’s rule. They are predicted to be water-soluble and to have high gastrointestinal absorption and bioavailability.

Scopoletin and umckalin showed higher lipophilicity than catechin, and gallic acid and showed the ability to cross the blood-brain barrier as indicated by their presence in the yolk of the brain or intestinal estimated permeation (BOILED-Egg) model (Figure S2) [42]. Only catechin is predicted to be effluated from the central nervous system by the P-glycoprotein.

### 2.6. Docking Study

Computational methods are increasingly used tools in drug discovery. They showed a potential role in the screening and development of several important drugs [43]. In this study, the four selected *P. sidoides* biomolecules were subjected to a computational docking study against several essential targets for SARS-CoV-2 viral replication and multiplication. These targets included the viral proteases; the papain-like protease (PLpro), and the main protease (Mpro) responsible for the proteolytic processing of the polyproteins that are translated from the viral RNA [22]. This proteolytic process is essential for the production of the functional proteins responsible for viral replication [23,24]. In addition to the viral proteases, SARS-CoV-2 RNA helicase (nsp13) plays an essential role in viral replication by unwinding duplex oligonucleotides into single strands. While, RNA-dependent RNA polymerase (RdRp, nsp12) is responsible for replicating the viral RNA genome [25]. So, they are among the most important therapeutic targets that could be used for inhibiting viral replication [26,27]. The ability of *P. sidoides* biomolecules for interfering with the interaction between the SARS-CoV-2 spike protein and the human angiotensin-converting enzyme 2 (ACE2) receptors, involved in the viral entry to the human cell [28,29] was also investigated.

Generally, the obtained results confirmed the presence of a strong relationship between the antiviral activity and the in-silico docking calculations of the studied *P. sidoides* biomolecules. The docking study demonstrated that scopoletin exhibited a good binding affinity to the tested SARS-CoV-2 targets that were higher or comparable to that of the co-crystallized ligands or standard inhibitors in most cases (Table 4).

The coumarin derivative, scopoletin showed good binding affinity towards RNA helicase, nsp13 (−6.5 Kcal/mol) which was greater than that of the co-crystalized ligand (−5.7 Kcal/mol). It also showed good binding affinity (−6.4 Kcal/mol) towards the interface region between the receptor-binding domain (RBD) of SARS-CoV-2 spike protein and the human cell angiotensin-converting enzyme 2 (ACE2) surface receptor indicating the probability of its interference with the viral entry to the human cells. Additionally, it demonstrated reasonable binding free energy (−5.2 Kcal/mol) with Mpro which is comparable to that of the co-crystalized ligand (−4.9 Kcal/mol), Table 4.

**Figure 4:**
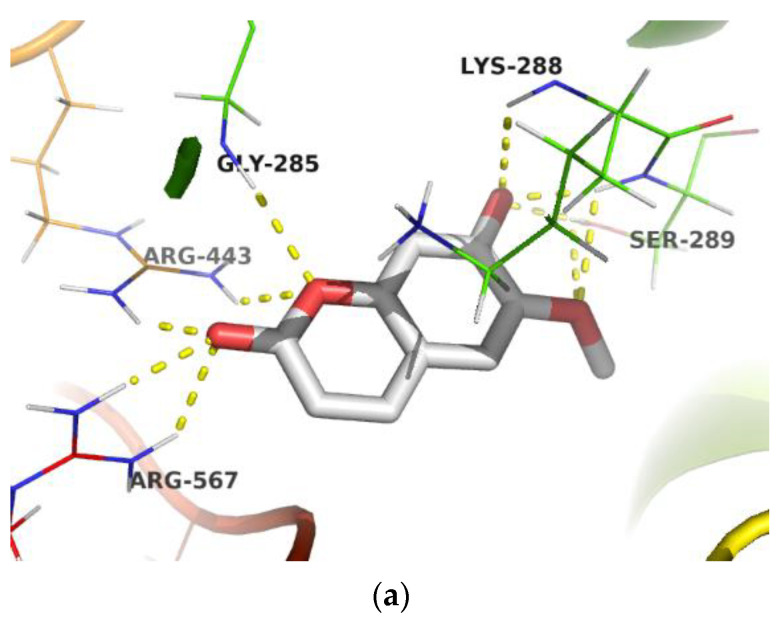

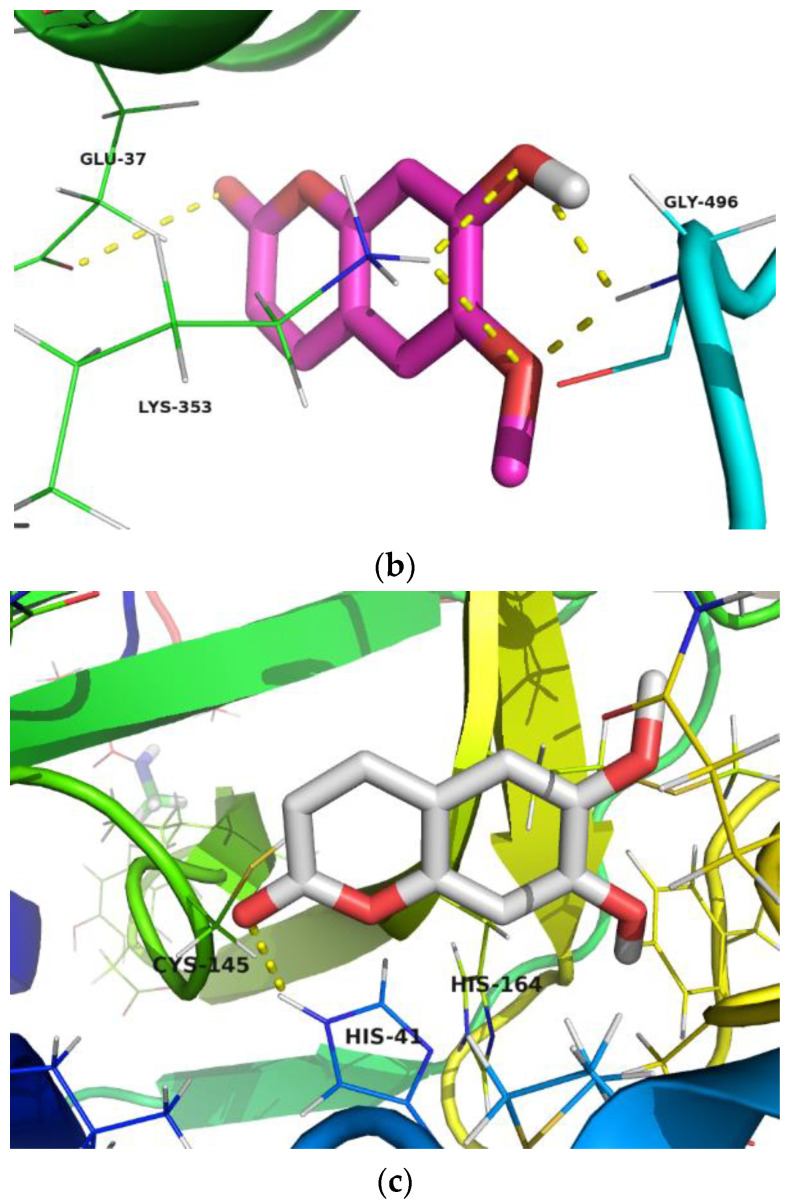
Molecular binding model of scopoletin within the active site of (**a**) the main protease (Mpro, PDB: 5R82); (**b**) within the interface region between the spike RBD (colored cyan) and human ACE2 (colored green), (PDB code: 6M0j); (**c**) within the active site of RNA helicase, nsp13 (PDB code: 5RL9) obtained by AutoDock Vina in PyRx 0.8 (Scripps Research, La Jolla, CA, USA).

Although umckalin showed close binding scores to that of scopoletin, visualization of the docking results indicated that scopoletin had better interaction with the amino acids important for protein activity. This may explain the higher in vitro antiviral activity of scopoletin in comparison to umckalin. Scopoletin formed hydrogen bonding with His-41 amino acid residue (Figure 4a) in the active pocket of the main protease (Mpro), which is reported to be involved in the Cys/His catalytic dyad essential for the proteolytic activity of Mpro [44]. It also showed hydrogen bonding with some amino acids essential for the interaction between the SARS-CoV-2 spike protein RBD (Gly-496), and the ACE2 surface receptor (Lys-353 and Glu-37), Figure 4b [29]. Remarkably, it showed hydrogen bonding with several amino acids, including Ser-289, Lys-288, and Arg-567, which are important for the NTPase activity of the RNA helicase [45], Figure 4c.

Although catechin demonstrated high docking scores towards the investigated protein targets as depicted in Table 4, it showed lower in vitro antiviral activity (IC_50_ 58.55 μM) than scopoletin (IC_50_ 17.79 μM). This may be attributed to its predicted active efflux by the P-glycoprotein and lower lipophilicity in comparison to scopoletin.

## 3. Materials and Methods

### 3.1. Chromatographic Procedures

#### 3.1.1. Solvents and Mobile Phases

All the reagents used in the study were of an HPLC grade. The acetonitrile for HPLC ≥ 99.9% was acquired from SIGMA-ALDRICH^®^ (Saint Louis, MO, USA). The methanol was of HPLC grade and purchased from Fisher Chemical (Waltham, MA, USA). The ethyl acetate was acquired from SIGMA-ALDRICH^®^ (Saint Louis, MO, USA). In addition, HPLC grade water was obtained from a Milli-Q ultrapure water system. We used orthophosphoric acid for HPLC 85–90% (Honeywell Fluka^TM^, Seelze, Germany) to adjust the pH. Finally, a 0.45 μm Nylon membrane HNWP filter was purchased from MERCK MILLIPORE^®^ Ltd. (Billerica, MA, USA).

#### 3.1.2. Samples

This experimental study utilized two brands of syrup phytopreparations containing *P. sidoides* tincture, including Kalobin^®^ (Marcyrl Pharmaceutical Industries, Cairo, Egypt) Batch No: 1944426, Expiry date: November 2022 and Umca^®^ (ABDIBRAHIM, Istanbul, Turki) Batch No: 2220420, Expiry date: March 2022, in addition to one brand of film-coated tablets (Umca^®^, ABDIBRAHIM, Istanbul, Turki) Batch No: 2480520, Expiry date: February 2025.

#### 3.1.3. Instrumentation and Chromatographic Conditions

In this study, the UFLC-SHIMADZU 1200 series system (Shimadzu Corporation, Santa Clara, CA, USA) was used to perform the chromatographic analysis of the samples. The system was attached to a binary pump, an online degasser, and the autosampler (SIL-20 A). The final separation was carried out using a 5 µm particle size XBridge^®^ C18 column (Milford, MA, USA) with the dimensions of 200 mm × 4.6 mm supplied by Waters, Ireland. The elution was isocratic at 1 mL/min with a mobile phase system of acetonitrile-water (20:80, *v*/*v*) adjusted to pH 3 by dropwise addition of 10% orthophosphoric acid. Finally, the mobile phase was filtered through 0.45 µm membrane filters and degassed by sonication for 15 min prior to use. The injection volume was 20 µL, and the flow rate was maintained at 1 mL/min with a total run time of 25 min at 30 °C. The liquid chromatographic analysis system was also equipped with a photodiode array detector (SPD—20 A) with a spectral setpoint of 210 nm. The system was connected to a computer running the LabSolutions software on a Microsoft Windows 7 operating platform.

#### 3.1.4. Analytical Standards

The four biomolecules required for this study were all purchased from SIGMA-ALDRICH^®^ (Taufkirchen, Germany) including umckalin (7-hydroxy-5,6-dimethoxycoumarin), HPLC determined purity ≥ 95.0%, CAS Number: 43053-62-9, scopoletin (7-hydroxy-6-methoxycoumarin), HPLC determined purity ≥ 97.0%, CAS Number: 92-61-5, (+)−catechin (syn. cianidanol), HPLC determined purity ≥ 99.0%, CAS Number: 154-23-4, and gallic acid (3,4,5-trihydroxybenzoic acid), HPLC determined purity ≥ 99.0%, CAS Number: 149-91-7.

#### 3.1.5. Preparation of the Test Solutions

To perform the intended analysis of this study, two different forms were considered, solution and tablet dosage forms. As for the solution form, Kalobin^®^ and Umca^®^ 20 mL-package were used. Each of the two solutions was processed by two different methods:

(a) Dilution of 1.1 mL solution, which contains roughly 100 mg of the dry root *P. sidoides* extract, in a 10 mL-volumetric flask. The dilution was carried out using methanol and the solution was subjected to sonication and filtration to obtain a 10 mg/mL solution.
(b) Liquid-liquid extraction (LLE) of a 20 mL tincture sample containing roughly 2000 mg of dry root *P. sidoides* extract with 3 × 20 mL of ethyl acetate. A rotary evaporator was then used to evaporate the combined ethyl acetate extracts to dryness at 45 °C. Finally, the resulting residue weighing 190.9 mg was dissolved in methanol and the volume was adjusted in a 5 mL-volumetric flask.

For the tablet dosage form of the plant extract, 10 Umca^®^ tablets containing 20 mg of dry *P. sidoides* root extract with an average weight of 0.403 g were crushed in a glass mortar. The obtained powder was equally divided into two portions that were processed by two different methods:

(a) The first portion (2.015 g) was extracted using a 10 mL methanol solution, sonicated for 5 min, and filtered. Finally, the volume of the solution is adjusted using a 10 mL-volumetric flask to obtain a 10 mg/mL concentration.
(b) The second portion (2.015 g) was suspended in 10 mL of water, sonicated, and extracted using 3 × 20 mL of ethyl acetate. Then, a rotary evaporator was used to evaporate the combined extracts to dryness at 45 °C. The produced residue was reconstituted in methanol and the volume was adjusted in a 5 mL-volumetric flask.

The final solution obtained from each procedure was filtered through a 0.45 µm membrane and the leading few mL of the filtrate were discarded. This procedure was repeated three times and injected in triplicate. Each time the peak area was calculated and recorded. The four biomolecules’ contents were calculated using the average and compared with the standards.

#### 3.1.6. Method Development and Validation

##### Preparation of the Standard Solution and the Quality Control Samples

Each of the four biomolecules’ reference solutions, namely gallic acid, catechin, scopoletin, and umckalin were diluted to a concentration of 1 mg/mL by dissolving 10 mg into 10 mL of methanol. The resulting solution was diluted again with methanol to produce a standard stock solution with a concentration of 100 µg/mL. This was, then, diluted once more with methanol to different levels to achieve concentrations in the interval of 0.1–1 µg/mL. Finally, further dilution of the four 100 µg/mL stock solutions with the same solvent was used to produce three quality control samples with the concentrations: 0.2 µg/mL (low quality control, LQC), 0.3 µg/mL (medium quality control, MQC), and 0.5 µg/mL (high quality control, HQC).

##### Specificity

HPLC chromatogram of the *P. sidoides* solutions and tablets was used to compare the UV spectra of the four biomolecules under the study. This procedure was repeated twice with samples of the solutions produced after acid (0.1 M HCl) and base (0.1 M NaOH) treatment of samples of the *P. sidoides* tincture [16]. The solution (2 mL) was added to the aqueous acid or base (2 mL) and the mixture was shaken for one hour at 25 °C. The mixture was then neutralized and injected.

##### Linearity

To prepare the standard calibration curves, five calibrators were utilized within the concentration interval of 0.2–1 µg/mL for gallic acid and catechin, and 0.1–1 µg/mL for scopoletin and umckalin. Linear least square (LLS) regression was used to interpolate the peak area with respect to the drug concentration curves. The accuracy of the regression for all the four biomolecules was measured employing the coefficient of determination ($R^{2}$) and the results were 0.9995, 0.9999, 0.9998, and 0.9995 for gallic acid, catechin, scopoletin, and umckalin, respectively.

##### Accuracy and Precision

To assess the accuracy and precision of the experimental procedure, the three QC samples with concentrations of 0.2, 0.3 and 0.5 µg/mL (low, medium, and high) were reproduced at three different time instances together with the five calibrators of the four standards. The precision was measured by the percentage relative standard deviation (% RSD), whereas the accuracy percentage was calculated from Equation (1):

(1) $$\%\;\mathrm{Accuracy}\;=\frac{\mathrm{Calculated}\;\mathrm{concentration}}{\mathrm{Nominal}\;\mathrm{concentration}}\times 100$$

##### Robustness

The robustness of the experimental chromatographic procedure was determined for different parameter values. All experiments performed in this study were repeated three times. Solutions with a concentration of 0.16 µg/mL were used to assess the stability of the procedure. The produced solutions were analyzed at four-time instances: immediately following the preparation, after 7 days, after 14 days, and after 30 days. For the latter three, the solutions were stored at a temperature of 4 °C. The obtained responses from all four experiments were plotted and compared. On the other hand, the stability of the samples was investigated by considering chromatographic analysis immediately following the preparation, and after 24 h of storage at the temperature of 25 °C.

### 3.2. Assay Procedure for the Determination of the Four Biomolecules in Kalobin, Umca Solutions, and Umca Tablets

Accurately weighed 10 mg portions of gallic acid, catechin, scopoletin, and umckalin reference standards were placed into 10 mL volumetric flasks and diluted with 10 mL of methanol to produce 1 mg/mL standard solutions. More diluted solutions with a concentration of 200 µg/mL were then obtained by mixing 5 mL of each reference standard with methanol in a 25 mL-volumetric flask. Then, 70 µg/mL working standard solutions were obtained by depositing 8.75 mL of each solution to a 25 mL-volumetric flask and completing the volume with acetonitrile-methanol (1:1 *v*/*v*). Finally, 20 µL portions of the working solutions were injected and the peak area was calculated each time. The corresponding chromatograms were recorded for the standard and the test solutions. Responses of the peak areas were obtained from the plots and concentration was calculated compared with the standard.

### 3.3. Evaluation of the In Vitro Inhibitory Activity against SARS-CoV-2

#### 3.3.1. The Used Cells and the Viral Strain

Vero E6 cells were obtained from the American Type Culture Collection (ATCC No. CRL-1586). The used SARS-CoV-2 strain, hCoV-19/Egypt/NRC-03/2020 (Accession Number on GSAID: EPI_ISL_430820) was isolated on 18 March 2020 from an oropharyngeal swab specimen obtained from a 34-year-old Egyptian woman [46]. All the experiments were executed according to the protocol approved by the ethics committee of the National Research Centre (NRC), Giza, Egypt (Approval number: NRC-20074).

#### 3.3.2. MTT Cytotoxicity Assay against Normal Cell Line (Vero E6)

The half-maximal cytotoxic concentration (CC_50_) of the test compounds was evaluated using the 3-(4,5-dimethylthiazol-2-yl)-2,5-diphenyltetrazolium bromide (MTT) assay with slight modifications [47] and according to the previously published procedure [48]. The percentage of cytotoxicity was measured compared to the untreated cells from Equation (2):

(2) $$\%\;\mathrm{cytotoxicity}=\frac{\mathrm{Absorbance}\;\mathrm{without}\;\mathrm{treatment}-\mathrm{Absorbance}\;\mathrm{with}\;\mathrm{treatment}}{\mathrm{Absorbance}\;\mathrm{without}\;\mathrm{treatment}\;}\times 100$$

Plotting% cytotoxicity versus the sample concentrations was used to calculate the concentration of the test compound that is required to reduce the cell viability by 50% (CC_50_).

#### 3.3.3. Cytopathic Effect (CPE) Inhibition Assay

The inhibitory concentration 50 (IC_50_) of the test compounds required to reduce the SARS-CoV-2-induced cytopathic effect on Vero E6 cells by 50% relative to the virus control was measured. The viral proliferation was assessed by visualization of the cytopathic effects on infected Vero E6 cells using crystal violet staining according to the previously published procedure [48]. The optical density of the produced color was measured at 570 nm using an Anthos Zenyth plate reader (Model: 200rt, Anthos Labtec Instruments, Heerhugowaard, The Netherlands).

#### 3.3.4. Statistical Analysis

The data obtained were analyzed using GraphPad Prism Software version 8 (San Diego, CA, USA). Data are shown as mean ± standard deviation (SD). One-Way ANOVA followed by Dunnett’s test was used to analyze the data. All concentrations of the tested samples showed statistical significance (*p* < 0.05) from the untreated cytopathic cells except for the lowest tested concentration of umckalin.

### 3.4. In Silico Studies

#### 3.4.1. Evaluation of Molecular and Pharmacokinetic Properties

The physicochemical and pharmacokinetics properties were investigated using the SwissADME online platform [49].

#### 3.4.2. Docking Studies

Molecular docking calculations were performed with AutoDock Vina [50] embedded in PyRx 0.8 software (Scripps Research, La Jolla, CA, USA). Docking was performed as described in the Supplementary Materials using crystal structures for the studied enzyme targets retrieved from RCSB-Protein Data Bank with the PDB codes displayed in Table S1. The docking results were visualized using Pymol Molecular Graphics System (Schrödinger, LLC, NY, USA) to view the docked protein-ligand interactions.

## 4. Conclusions

A new HPLC-UV analytical method has been developed and validated for separating and detecting four selected biomolecules (i.e., gallic acid, catechin, scopoletin, and umckalin) of *P. sidoides* root extract in different commercial dosage forms. The efficiency of the proposed method was verified by an assay testing procedure considering the commercial forms: Kalobin^®^ and Umca^®^ solutions and Umca^®^ tablets. The developed method was accurate, precise, fast, and superior in multiple respects to other previously reported methods of the analysis of the *P. sidoides*’ biomolecules. Unlike existing methods, the developed method can separate all investigated biomolecules simultaneously in their pure form and in different dosage forms. These results indicated that the proposed method can be successfully employed for the routine analysis of the four investigated biomolecules in bulk and commercial formulations containing this plant extract. Out of the four tested biomolecules, scopoletin, in addition to *P. sidoides* DC. root extract, showed remarkable in vitro antiviral activity against SARS-CoV-2 using cytopathic effect (CPE) inhibition assay on Vero E6 cells. However, the chemotaxonomic marker “umckalin” exhibited weak antiviral activity against SARS-CoV-2.

## Figures and Tables

**Table 1:** Analytical performance data for the determination of the studied compounds by the proposed method.

Parameter *	Gallic Acid	Catechin	Scopoletin	Umckalin
Concentration range (µg/mL)	0.2–1	0.2–1	0.1–1	0.1–1
Correlation coefficient (r)	0.9995	0.9999	0.9998	0.9995
Slope	129,746.6	164,166.5	162,561.9	188,550.2
Intercept	1655.6	2039.8	2223.6	3046.9
$S_{y/x}$, S.D. of the residuals	1059.3	670.3	827.0	1456.5
$S_{a}$, S.D. of the intercept	943.78	597.28	589.57	1038.3
$S_{b}$, S.D. of the slope	1700.6	1076.2	1159.9	2042.8
S.D.	1.95	1.08	1.87	2.12
%RSD ^a^	1.95	1.08	1.86	2.11
%Error ^b^	0.873	0.484	0.762	0.866
LOD ^c^ (µg/mL)	0.024	0.012	0.012	0.018
LOQ ^d^ (µg/mL)	0.073	0.036	0.036	0.055

* All determinations were conducted at 210 nm. ^a^ Percentage relative standard deviation. ^b^ Percentage relative error. ^c^ Limit of detection. ^d^ Limit of quantitation.

**Table 2:** Statistical evaluation of the precision and accuracy for the analysis of the biomolecules in their pure forms by the proposed method.

Responses	Concentrations (μg/mL) *			
	Gallic Acid	Catechin	Scopoletin	Umckalin
**Response at 0.2 µg/mL**	**Concentration:** **0.2 µg/mL**			
	28,551	34,214	34,084	40,077
	28,740	34,350	34,079	40,223
	28,340	34,153	34,090	40,122
**X**	28,543.67	34,239.00	34,084.33	40,140.67
**SD**	200.10	100.85	5.51	74.77
**%RSD**	0.70	0.29	0.02	0.19
Conc. Found * **(µg/mL)**	0.204	0.196	0.196	0.196
**% Found**	102.10	98.20	98.00	98.40
**Response at 0.3 µg/mL**	**Concentration: 0.3 µg/mL**			
	40,552	51,184	51,610	60,560
	41,019	51,435	51,633	60,443
	40,391	51,099	51,621	60,492
**X**	40,654.00	51,239.33	51,621.33	60,498.33
**SD**	326.19	174.70	11.50	58.76
**%RSD**	0.80	0.34	0.02	0.10
Conc. Found * **(µg/mL)**	0.307	0.298	0.304	0.304
**% Found**	102.33	99.53	101.30	101.57
**Response at 0.5 µg/mL**	**Concentration: 0.5 µg/mL**			
	65,527	75,560	72,072	83,973
	65,256	75,118	72,253	84,031
	64,322	75,233	72,165	83,992
**X**	65,035.00	75,303.67	72,163.33	83,998.67
**SD**	632.17	229.32	90.51	29.57
**%RSD**	0.97	0.30	0.13	0.04
Conc. Found * **(µg/mL)**	0.491	0.505	0.504	0.507
**% Found**	98.24	101.00	100.80	101.56
$\bar{X}$ **± SD**	100.89 ± 2.2	99.57 ± 1.4	100.03 ± 1.7	100.51 ± 1.8

* Each result is the average of three separate determinations. X = average SD = standard deviation %RSD = Percentage relative standard deviation Conc. Found = concentration found % Found = Percentage found. $\bar{X}$ = Mean Accuracy.

**Table 3:** The 50% cytotoxic concentration (CC_50_) on normal Vero E6 cells, the 50% inhibitory concentration (IC_50_) against SARS-CoV-2-induced cytopathic effect in Vero E6 cells by *P. sidoides* root extract ± S.E.M., and the assay results of gallic acid, catechin, scopoletin, and umckalin content in Kalobin^®^, Umca^®^ solutions (S) and Umca^®^ tablets (T).

Test Sample	CC_50_ (μM) ^a^	IC_50_ (μM) ^b^	Selectivity Index (SI)	Concentration of Investigated Biomolecules (μg/mL) **					
				Kalobin^®^-S		Umca^®^-S		Umca^®^-T	
				Ethyl Acetate	Methanol	Ethyl Acetate	Methanol	Ethyl Acetate	Methanol
**Gallic acid**	439.7 ± 0.065	96.41 ± 0.030	4.6	89.54	22.00	188.71	98.91	146.66	23.72
**Catechin**	52.76 ± 0.079	58.55 ± 0.088	0.9	14.05	10.96	39.09	0	11.26	0
**Scopoletin**	253.1 ± 0.45	17.79 ± 0.91	14.1	14.86	0	6.31	0	27.19	0
**Umckalin**	164.1 ± 0.54	311.6 ± 0.043	0.5	121.07	3.09	79.46	2.86	126.33	4.54
***P. sidoides* root extract**	87.25 ± 0.093 ^c^	13.79 ± 0.034 ^c^	6.3						
Chloroquine *	377.7	22.7	16.64						
Hydroxychloroquine *	356	32.8	10.85						

^a^ The cytotoxicity was determined using an MTT assay and values were calculated by nonlinear regression analysis using the GraphPad Prism software version 8 (San Diego, CA, USA). ^b^ Values were calculated by plotting the log of the inhibitor’s concentrations versus normalized response (variable slope) using the GraphPad Prism software version 8 (San Diego, CA, USA). ^c^ Values are measured in μg/mL for the crude extract. * The IC_50_ and CC_50_ values of the antiviral positive controls (chloroquine and hydroxychloroquine) were reported by the same laboratory [38]. ** Concentrations are expressed as the mean of triplicate injection by the proposed HPLC method.

**Table 4:** Docking scores of *Pelargonium sidoides* biomolecules against the SARS-CoV-2 main therapeutic targets using AutoDock Vina in PyRx 0.8.

Compound	Binding Energy (Kcal/mol)				
	Mpro	PLpro	Nsp13	RdRp	The Interface of RBD of Spike Protein with Its Human ACE2 Receptor
**Catechin**	−7.2	−6.1	−7.2	−6.4	−7.7
**Gallic acid**	−5.1	−4.5	−5.7	−5.4	−6.4
**Umckalin**	−5.3	−4.8	−6.6	−5.4	−6.3
**Scopoletin**	−5.2	−4.9	−6.5	−5.8	−6.4
**Reference inhibitor**	−4.9	−6.7	−5.7	−8.9	−6.6

## Data Availability

Data is contained within the article and Supplementary Materials.

## Supplementary Materials

The following supporting information can be downloaded at: https://www.mdpi.com/article/10.3390/ph15101184/s1, Table S1. PDB codes of the crystal structures and grid box coordinates for the enzymes used in the docking study; Table S2. The in-silico predicted physicochemical and ADME properties of *Pelargonium sidoides* biomarkers; Figure S1. Calibration curve of: (a) gallic acid, (b) catechin, (c) scopoletin, and (d) umckalin; Figure S2. BOILED-Egg representation of lipophilicity and polarity of *Pelargonium sidoides* biomarkers.
