# Supplementary material for: Pelargonium sidoides Root Extract: Simultaneous HPLC Separation, Determination, and Validation of Selected Biomolecules and Evaluation of SARS-CoV-2 Inhibitory Activity
Source: Pharmaceuticals (Basel). 2022 Sep 23;15(10):1184. doi: 10.3390/ph15101184 (PMC9610410; doi:10.3390/ph15101184)
Supplement: Supplementary file 1 [file pharmaceuticals-15-01184-s001.zip › pharmaceuticals-1891211-supplementary.pdf]

Supplementary material

Pelargonium Sidoides Root Extract: Simultaneous HPLC Separation, Determination, and Validation of Selected Biomolecules and Evaluation of SARS-Cov2 Inhibitory Activity

Manal A. Alossaimi[1], May A. Alzeer[2], Fatma M. Abdel Bar[2,3]*, Mai H. ElNaggar[4]

[1] Department of Pharmaceutical Chemistry, College of Pharmacy, Prince Sattam Bin Abdulaziz University, Al-Kharj 11942, Saudi Arabia

[2] Department of Pharmacognosy, College of Pharmacy, Prince Sattam Bin Abdulaziz University, Al-Kharj 11942, Saudi Arabia

[3] Department of Pharmacognosy, Faculty of Pharmacy, Mansoura University, 35516, Egypt

[4] Department of Pharmacognosy, Faculty of Pharmacy, Kafrelsheikh University, 33516, Kafrelsheikh, Egypt

*Author for correspondence: Fatma M. Abdel Bar; Tel: +966545403617

E-mail: fatma_maar@yahoo.com; f.abdelbar@psau.edu.sa

ABSTRACT

This study aimed to establish a validated HPLC-UV analytical method for the determination of gallic acid, catechin, scopoletin, and umckalin in phytoformulations containing *P. sidoides*. Also, to assess the anti-SARS-CoV2 effect of *P. sidoides* and these biomolecules *in vitro*. An HPLC-UV method was developed and verified by testing the commercial forms, Kalobin® and Umca®. It revealed low detectable scopoletin and high umckalin levels. *P. sidoides* exhibited a significant reduction of SARS-CoV2-induced cytopathic effect in Vero E6 cells (IC_{50} 13.79 μg/mL and selectivity index, SI 6.3). Whereas scopoletin showed a remarkable anti-SARS-CoV2 activity with better selectivity (IC_{50} 17.79 μg/mL and SI 14.22). An *in-silico* prediction of the drugability indicated that the studied biomolecules are under the acceptable norms of Lipinski's rule, water-soluble, and showed high GIT absorption and bioavailability. Docking study towards the essential molecular targets for viral replication and entry of SARS-CoV2 indicated good binding affinity of scopoletin (-6.4 Kcal/mol) towards the interface region between the SARS-CoV2 spike protein RBD and the ACE2 surface receptor indicating the probability of interference with the viral entry to the human cells and showed H-bonding with His-41 in the active site of the main protease which may explain its high antiviral activity.

Keywords: COVID-19; HPLC-UV; Quality control of Umckaloabo; *Pelargonium sidoides* root extract; Umckalin.

Docking studies

The docking sites were defined by establishing a grid box at the geometrical center of the co-crystallized ligand present in the used PDB structure or the main amino acids involved in the enzyme activity with the dimensions of 25 x 25 x 25 Å. The coordinates X, Y and Z from the centre of the grid box in case of each enzyme docking and the used reference inhibitor are mentioned in Table S1.

Table S1. PDB codes of the crystal structures and grid box coordinates for the enzymes used in the docking study.

SARS-CoV2 enzymes	PDB codes	Grid box X, Y and Z coordinates	Reference inhibitor
Main protease (Mpro)	5R82	10.8206, -0.4913 and 22.7101	**RZS:** 6-(ethylamino)pyridine-3-carbonitrile, the co-crystallized ligand.
Papain-like protease (PLpro)	6W9C	-44.5243, 4.7841and 37.2356	**GRL:** 5-amino-2-methyl-N-[(1R)-1-naphthalen-1-ylethyl]benzamide, a standard ligand inhibitor for PLPro.
RNA helicase	5RL9	-19.1598, 32.4786 and -24.0935	**UR7:** 1-(3-fluoro-4-methylphenyl)methanesulfonamide, the co-crystallized ligand.
RNA-dependent RNA polymerase (RdRp)	7D4F	124.5678, 133.3852 and 142.8610	**H3U:** 8-(3-(3-aminobenzamido)-4-methylbenzamido)naphthalene-1,3,5-trisulfonic acid, the co-crystallized ligand.
The interface of RBD of spike protein with its human ACE2 receptor	6M0j	-38.3612, 24.7032, 6.2665	**Emodin**, a standard ligand inhibitor.

Table S2. The *in-silico* predicted physicochemical and ADME properties of *Pelargonium sidoides* biomarkers

	**Catechin**	**Gallic acid**	**Umckalin**	**Scopoletin**
Molecular weight	290.27 g/mol	170.12 g/mol	222.19 g/mol	192.17 g/mol
Molecular formula	$C_{15}H_{14}O_6$	$C_7H_6O_5$	$C_{11}H_{10}O_5$	$C_{10}H_8O_4$
Lipophilicity (logP)	0.85	0.21	1.51	1.52
Water solubility	Soluble	Soluble	Soluble	Soluble
GIT absorption	High	High	High	High
BBB permeability	No	No	Yes	Yes
Bioavailability score	0.55	0.56	0.55	0.55
H-bond (donors/acceptors)	5/6	4/5	1/5	1/4
Bioavailability Radar figures	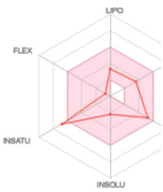	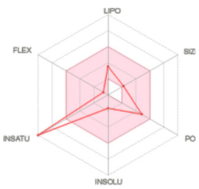	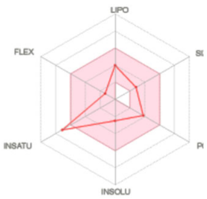	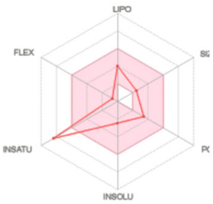

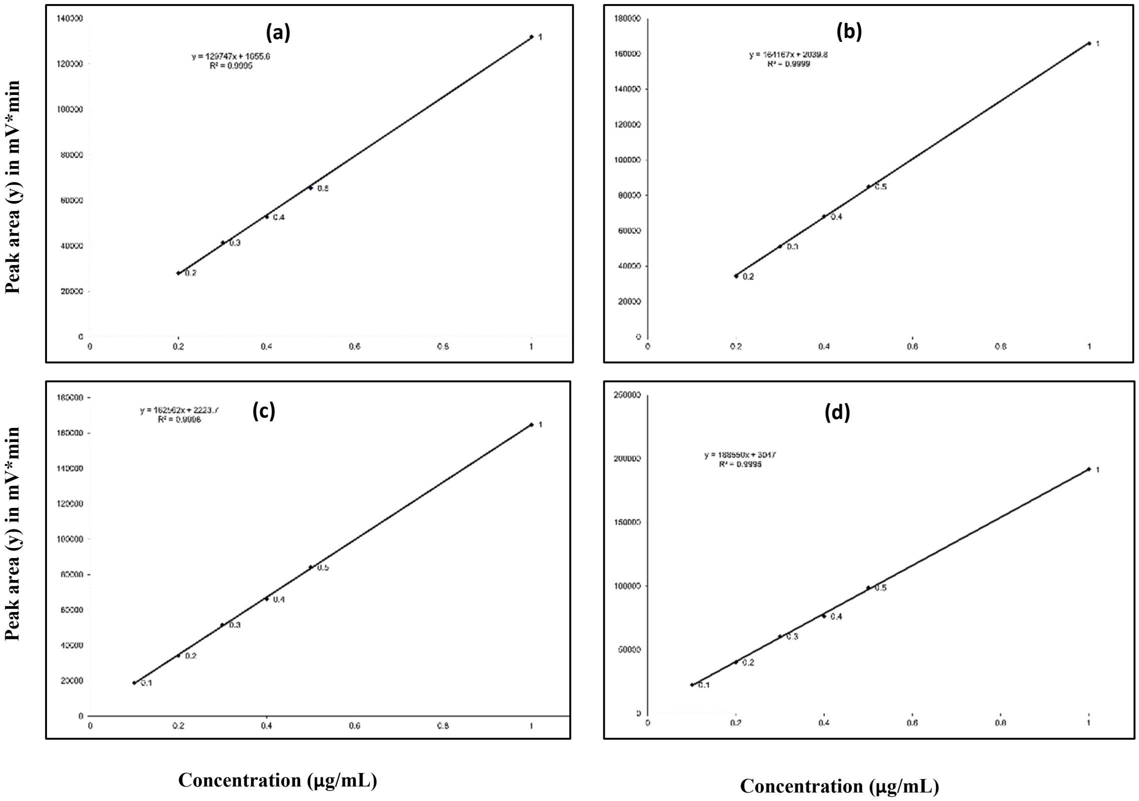

Figure S1. Calibration curve of: (a) gallic acid, (b) catechin, (c) scopoletin, and (d) umckalin.

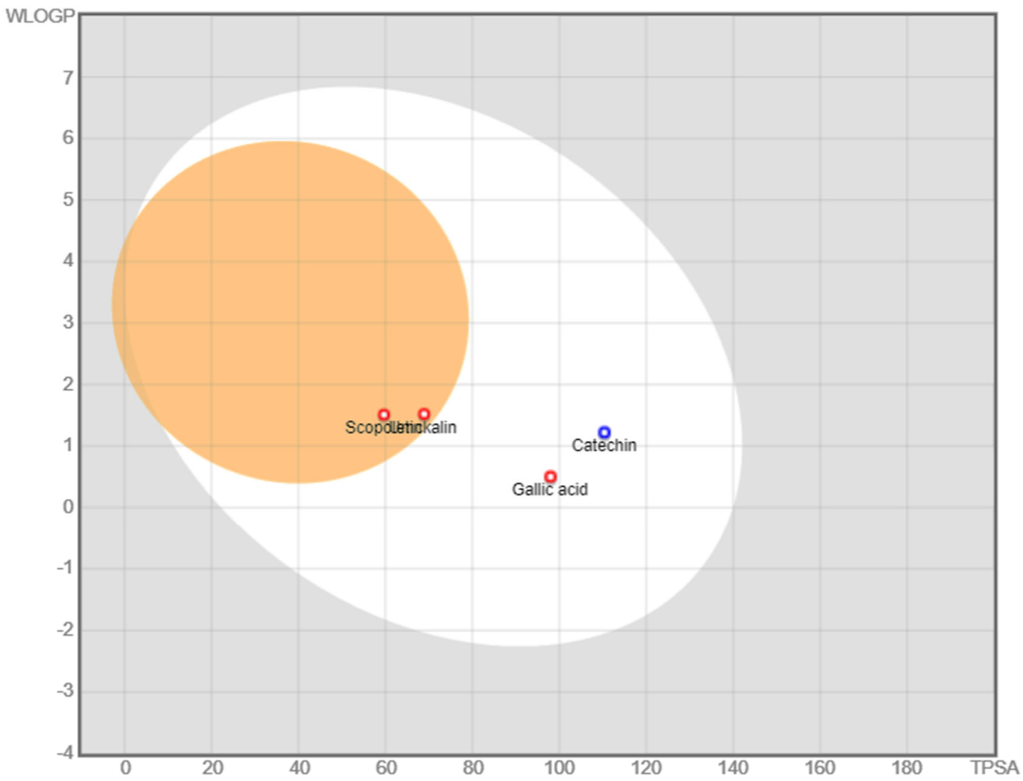

Figure S2. BOILED-Egg representation of lipophilicity and polarity of *Pelargonium sidoides* biomarkers.
